# Feasibility of implementing an intervention in general practice for deprescribing of glucose-lowering medication in overtreated elderly

**DOI:** 10.1093/fampra/cmaf064

**Published:** 2025-08-26

**Authors:** Charlotte Andriessen, Marieke T Blom, Beryl A C E van Hoek, Anna W de Boer, Petra Denig, Ron Herings, Angela de Rooij-Peek, Rob J van Marum, Jacqueline G Hugtenburg, Daniël van Raalte, Liselotte van Bloemendaal, Giel Nijpels, Marjan J Westerman, Rimke C Vos, Petra J M Elders

**Affiliations:** Department of General Practice, Amsterdam University Medical Center, Meibergdreef 15, 1105 AZ Amsterdam, The Netherlands; Amsterdam Public Health Research Institute, 1105 AZ Amsterdam, The Netherlands; Department of General Practice, Amsterdam University Medical Center, Meibergdreef 15, 1105 AZ Amsterdam, The Netherlands; Amsterdam Public Health Research Institute, 1105 AZ Amsterdam, The Netherlands; Department of General Practice, Amsterdam University Medical Center, Meibergdreef 15, 1105 AZ Amsterdam, The Netherlands; Amsterdam Public Health Research Institute, 1105 AZ Amsterdam, The Netherlands; Department of Public Health and Primary Care/Health Campus The Hague, LUMC, 2333 ZD Leiden, The Netherlands; Department of Clinical Pharmacy and Pharmacology, University of Groningen, University Medical Center Groningen, 9700 AB Groningen, The Netherlands; PHARMO Institute for Drug Outcomes Studies, 3528 AE Utrecht, The Netherlands; Diabetesvereniging Nederland, 3833 LD Leusden, The Netherlands; Department of Geriatric Medicine, Jeroen Bosch Hospital, 5223 GZ ‘s-Hertogenbosch, The Netherlands; Department of Clinical Pharmacology, Jeroen Bosch Hospital, 5223 GZ ‘s-Hertogenbosch, The Netherlands; Department of Elderly Care Medicine, Amsterdam University Medical Center, 1081 HV Amsterdam, The Netherlands; Department of Clinical Pharmacology and Pharmacy, Amsterdam University Medical Center, 1081 HV Amsterdam, The Netherlands; Department of Internal Medicine, Diabetes Center, Amsterdam University Medical Center, 1081 HV Amsterdam, The Netherlands; Department of Internal Medicine—Geriatrics, Amsterdam University Medical Center, 1081 HV Amsterdam, The Netherlands; Department of General Practice, Amsterdam University Medical Center, Meibergdreef 15, 1105 AZ Amsterdam, The Netherlands; Amsterdam Public Health Research Institute, 1105 AZ Amsterdam, The Netherlands; Department of Epidemiology and Data Science, Amsterdam University Medical Center, 1105 AZ Amsterdam, The Netherlands; Department of Public Health and Primary Care/Health Campus The Hague, LUMC, 2333 ZD Leiden, The Netherlands; Department of General Practice, Amsterdam University Medical Center, Meibergdreef 15, 1105 AZ Amsterdam, The Netherlands; Amsterdam Public Health Research Institute, 1105 AZ Amsterdam, The Netherlands

**Keywords:** quality improvement, implementation, general practice, type 2 diabetes, hypoglycaemia, elderly

## Abstract

**Background:**

Elderly patients with Type 2 diabetes (T2D) are frequently overtreated with glucose-lowering medication.

**Objective:**

This feasibility study evaluated the implementation of a deprescribing programme (DPP) for general practices, consisting of education, a patient selection tool, practice visits, and an expert support panel, before scaling it in a randomized controlled trial.

**Methods:**

Quantitative evaluation included the number of patients with T2D eligible for deprescribing using medical records and study progress data. Qualitative evaluation entailed the analysis of minutes made during training, and interviews with health care providers (HCPs). The extended normalization process theory guided analysis.

**Results:**

In 10 practices, 55 out of 65 eligible patients were deprescribed glucose-lowering medication, with 22 restarts. Most execution steps were perceived as the practice nurse's responsibility, whereas the general practitioner needed to approve the deprescribing. Practice nurses found the educational training, including peer-to-peer sessions and practice visits, supportive of integrating deprescribing into practice. DPP procedures and tasks not part of the regular care process were not consistently performed. The DPP was adapted to minimize study tasks for HCPs and align study procedures to existing routine procedures.

**Conclusion:**

Implementation of a DPP in general practice requires education, practice visits, and alignment of DPP components to regular care.

Key messages

- Practice nurses are key to deprescribing diabetes medication in general practice.
- Successful implementation requires close alignment to existing routine procedures.
- Practice visits by research assistants can facilitate the implementation process.

## Introduction

Approximately 20% of elderly patients with Type 2 diabetes (T2D) in the Netherlands are overtreated with insulin and/or sulphonyl urea derivatives, of whom most are treated in general practice [1, 2]. Overtreatment leads to an elevated risk of hypoglycaemia, which can result in falls, hospitalization, and a reduced quality of life [3–9]. In the guideline of the Dutch College of General Practitioners (Nederlands Huisartsen Genootschap; NHG), less stringent HbA1c targets are recommended for elderly T2D patients compared with younger patients [10]. Following this guideline, deprescribing of insulin and/or sulphonyl urea derivatives potentially causing hypoglycaemia can be considered in frail elderly T2D patients [11]. Nevertheless, the implementation of deprescribing these glucose-lowering medications is difficult [12]. Barriers to deprescribing include a lack of time of health care providers [HCPs, i.e. practice nurses (PNs) and general practitioners (GPs)], a lack of perceived need to change medication in a-symptomatic patients, a fear of patients to reduce or discontinue their medication, a lack of knowledge on medication reduction in PNs (∼licensed practical nurses), and the involvement of different stakeholders in the treatment of patients [13–15]. An additional barrier entails the lack of randomized-controlled trials (RCTs) that have been performed on the safety of deprescribing glucose-lowering medication in elderly patients [16].

We developed a deprescribing programme (DPP) to promote deprescribing glucose-lowering medication in general practices so that the safety of deprescribing could be assessed in an RCT: the OMED2 study (Optimization of Medication in Elderly with Diabetes). By providing education and support with deprescribing, we hypothesized that the DPP would facilitate deprescribing in general practice and would increase awareness of HCPs for patients receiving more medication than recommended. Before the OMED2 study, we conducted a study to assess the feasibility of implementing the DPP and concomitant study procedures in general practice. Here, we report the results of this feasibility study and provide information on adaptations made to the DPP to evaluate its impact in an RCT.

## Materials and methods

The feasibility study was conducted in general practices between April 2021 and June 2022. Most T2D-care in the Netherlands is performed in general practices by a PN under the supervision of a GP. The study had a follow-up period of 6 months. During follow-up, the programme was continuously evaluated and adjusted. The Standards for Quality Improvement Reporting Excellence guided the content of the research report [17].

### Deprescribing programme

**Figure 1.**
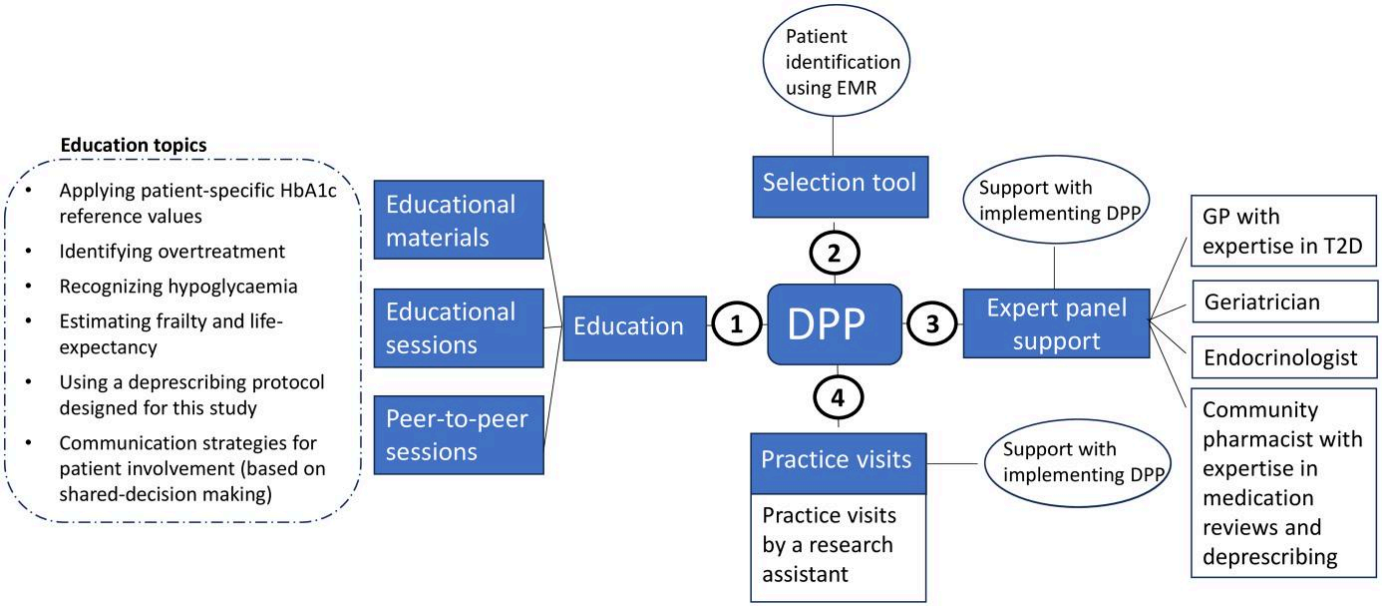
Elements of the DPP to facilitate deprescribing in general practice. Content of DPP based on Refs. [18, 19]. Abbreviations: DPP, deprescribing programme; EMR, electronic medical record; PN, practice nurse; GP, general practitioner.

**Figure 2.**
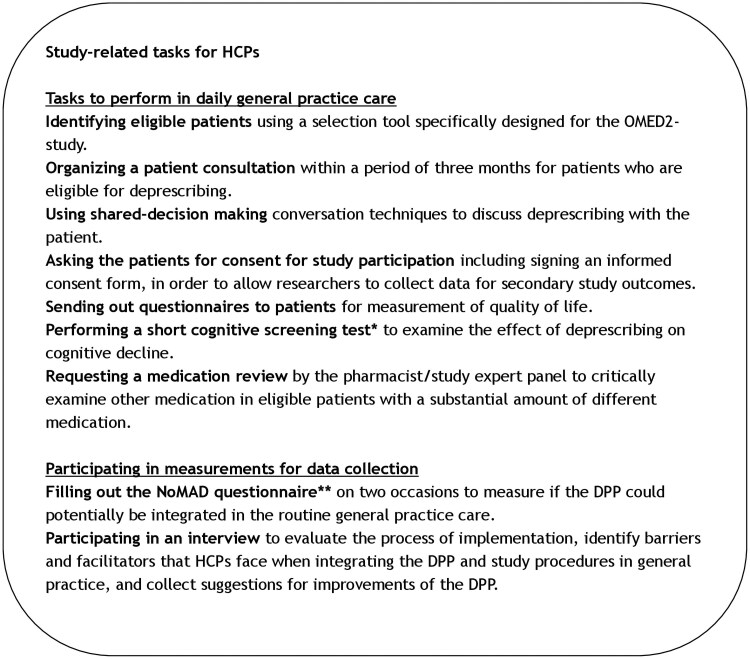
Study-related tasks for HCPs to study the implementation of the deprescribing programme. *Mini-Cog^©^ examination [18]. **NoMAD questionnaire: Normalization MeAsure Development [20]. Abbreviations: HCP, healthcare professional; OMED2, Optimization of Medication in Elderly with Diabetes; DPP, deprescribing programme.

Components of the DPP are depicted in Fig. 1. The DPP aimed to promote deprescribing of glucose-lowering medication by HCPs in overtreated elderly (≥70 years) patients with T2D. Definition of overtreatment and content of the DPP were based on the NHG-guideline [10, 18]. Briefly, patients who used sulphonyl urea derivatives and/or insulin, aged ≥70 years, and with HbA1c < 54 mmol/mol (7.1%) were considered overtreated. At the start of the intervention, all HCPs followed one educational session on deprescribing. A second educational session was organized for PNs. PNs were required to follow at least one peer-to-peer session, during which case studies on deprescribing were discussed under the supervision of a T2D expert GP. Furthermore, HCPs could request a practice visit from a research assistant to support them with implementing the DPP and the deprescribing protocol. Study-related tasks that were asked of HCPs and were on top of healthcare tasks are shown in Fig. 2.

### Recruitment of general practices and data collection

General practices were recruited by the Department of General Practice of Amsterdam University Medical Center and the Department of Public Health and Primary Care of Leiden University Medical Center. Data were extracted from the electronic medical records (EMR) of each general practice at the start of the study and after 6 months of follow-up. During the training, minutes were made which documented how the DPP was received by the HCPs and the questions, suggestions, and comments that were raised. The number of informed consent forms, Mini-Cog^©^ examinations performed, and medication reviews performed were documented by the research team. Semi-structured interviews with GPs and PNs were performed to gain insight into their experiences with the DPP and to improve the integration of the DPP. A topic list (Supplementary material) was constructed based on the expertise of the research team and using sensitizing concepts. The list entailed themes of the extended normalization process theory (ENPT), which is a commonly used framework to evaluate the implementation of innovations [20].

#### Quantitative feasibility outcomes

EMR data were used to determine how many patients were eligible for deprescribing, how many patients received deprescribing of glucose-lowering medication, and how many patients restarted their medication after deprescribing. Deprescribing was defined as any reduction in treatment intensity of sulphonyl urea derivatives and/or insulin, including discontinuing these treatments. Other quantitative outcomes included the number of completed informed consent forms from patients, Mini-Cog^©^ examinations performed, and medication reviews performed.

#### Qualitative feasibility outcomes

The training minutes and the interviews' transcripts were coded to assess barriers and facilitators of implementation in line with the thematic content analysis approach of Braun and Clarke [21]. The information was summarized to explore the feasibility of implementing the DPP in general practice. Each transcript was analysed by at least two researchers (B.A.C.E.v.H., L.v.B., and P.J.M.E.). In each analysis step, themes from the ENPT were identified. Researchers remained open to themes outside of the ENPT that were important for the integration of the DPP. With the software program MAXQDA (VERBI Software, Marburg, Germany), the transcript was marked with ENPT code labels. Comments were added to interesting segments outside of the ENPT.

### Ethics

The Medical Ethical Testing Committee of Amsterdam UMC declared that the study did not fall under the Medical Research Involving Human Subjects Act (WMO). The OMED2 study is registered at the ISRCTN registry (ISRCTN50008265). All participating general practices gave permission for data-sharing and signed a contract. Additional informed consent was obtained from HCPs prior to interviews. Patients signed informed consent if they were willing to fill out questionnaires.

## Results

Ten practices (six from Amsterdam and four from the area of Leiden) participated in the feasibility study. Using the EMR-selection tool, 65 patients were eligible for deprescribing, of which 55 patients were deprescribed (Table 1). From these 55 patients, 22 patients (40%) restarted with their medication. For 21 patients, the medication was increased to the original dose, whereas for 1 patient, the medication was increased with a lower dose than before deprescribing.

**Table 1.** Characteristics of deprescribing and frequency of study measurements per general practice.

General practice	P1	P2	P3	P4	P5	P6	P7	P8	P9	P10	Total^a^
Pt identified by selection tool, *n*	20	10	21	15	13	4	15	2	6	17	123
Pt eligible for deprescribing, *n* (% eligible from list)	6 (30%)	8 (80%)	9 (43%)	10 (66%)	4 (31%)	5^b^ (125%)	5 (33%)	6^b^ (300%)	2 (33%)	10 (59%)	65 (53%)
Medication reviews performed, *n*	5	8	9	10	4	5	5	7	2	10	65
Pt deprescribed, *n* (% eligible pt deprescribed)	5 (83%)	7 (88%)	6 (67%)	9 (90%)	3 (75%)	4 (80%)	5 (100%)	6 (100%)	2 (100%)	8 (80%)	55 (86%)
Pt restarted medication, *n* (% restarted)	3 (60%)	5 (71%)	3 (50%)	5 (56%)	0 (0%)	2 (50%)	0 (0%)	1 (17%)	0 (0%)	3 (38%)	22 (40%)
First Mini-Cog^©^ examination performed, *n*	2 (33%)	3 (38%)	1 (11%)	3 (30%)	2 (50%)	1 (20%)	1 (20%)	4 (66%)	1 (50%)	4 (40%)	22 (34%)
Second Mini-Cog^©^ examination performed, *n*	0	0	0	2 (20%)	0	0	0	1 (17%)	0	1 (10%)	4 (6%)

P, practice; Pt, patient; *n*, number of patients.

^a^Total of all practices combined.

^b^This practice found more patients eligible for deprescribing who were not on the list of the selection tool because time had elapsed between the generation of the patient list and the start of the programme, and therefore, new patients were identified.

In the 13 interviews that were performed (6 GPs and 7 PNs), both GPs and PNs indicated that the study procedures and the process of deprescribing, including administrative work and the assessment of a patient’s eligibility for deprescribing, were the responsibility of the PN. Most HCPs felt that deprescribing needed to be discussed with and approved by the GP. Half of the interviewed GPs indicated that study participation took too much time, whereas one GP indicated that it did not cost time since the PN executed all the work. From the interviews and minutes, 11 barriers and 4 facilitators for implementing the DPP (and study procedures) were identified (Table 2). Results are described according to the following topics: (i) beliefs of HCPs towards deprescribing and content of the education, (ii) integration of the DPP in general practice, and (iii) the incorporation of study-related tasks within routine practice care.

**Table 2.** Barriers and facilitators for implementation of the DPP informed by ENPT.

	Constructs and themes	Barriers (−) and facilitators (+)
Capability	The capability of HCPs to use components of the DPP within the general practice context. Themes belonging to this construct: Workability, which reflects, e.g. the user-friendliness of the selection tool Integration, which reflects, e.g. the effort of the HCP to integrate the DPP components in routine practice care	*Workability* Tasks to perform in daily general practice care (e.g. Mini-Cog^©^ examination, medication review) were mentioned to be too time-consuming for PNs (−) GPs mentioned that having only a few patients on the list who were eligible for deprescribing made the DPP easy to perform (+) *Integration* PNs indicated to experience difficulties in performing the shared decision-making process that was explained and demonstrated in the training (−) GPs and PNs viewed identifying patients eligible for deprescribing via selection tool as too time-consuming (−)
Capacity	The capacity of the social system of general practice to incorporate the DPP. Themes belonging to this construct: Social roles, which reflects, e.g. the person initiating the deprescribing Material resources, which reflects, e.g. the quality of the content of the educational materials Cognitive resources, which reflects, e.g. the knowledge level on deprescribing of the GP	*Social roles* PNs were not experienced in asking for study participation via informed consent (−) *Material resources* HCPs asked several questions about the deprescribing schedule during the training (−) Most GPs and PNs considered the educational sessions very informative (+) GPs and PNs found the peer-to-peer sessions useful for discussing challenging case studies (+) PNs indicated that they deemed the practice visits very encouraging (+) *Cognitive resources* PNs and GPs found it difficult to estimate life expectancy and frailty of a patient (−)
Potential	How much potential there is to implement the DPP in general practice. Themes belonging to this construct: Individual attitudes, which reflects, e.g. the intrinsic motivation of the PN for deprescribing	*Individual attitudes* Three PNs indicated that they already worked according to guidelines on deprescribing and felt no need for further education (−) Two GPs indicated not to be motivated to participate in DPP actively (−) Four GPs and three PNs deemed deprescribing medication in older patients important for achieving better quality of care and were enthusiastic about the goal of the programme (+)
Contribution	The implementation of the DPP depends on continuous contributions of the involved HCPs. Themes belonging to this construct: Coherence/sense-making, which reflects, e.g. the ability of the PN to apply the educated deprescribing steps in overtreated patients Cognitive participation, which reflects, e.g. the discussion the PN has with the GP when deprescribing a patient	*Coherence/sense-making* GPs and PNs raised concerns about the rise in HbA1c when following the deprescribing scheme from the education (based on NHG-guidelines) (−) *Cognitive participation* HCPs experienced that other specialists involved in patient care (e.g. cardiologist/pharmacist) recommended increasing glucose-lowering medication instead of deprescribing (−)

DPP, deprescribing programme; HCPs, healthcare professional; GP, general practitioner; PN, practice nurse.

### Beliefs towards deprescribing and the content of education

Almost all HCPs believed that deprescribing medication in overtreated elderly patients improves patient care and expressed motivation to participate in the study.

*I think that many people use too much medication, so I agree with less medication and in a different way, so it [the DPP] fits with my way of working. (PN1)*

Several HCPs expressed reluctance towards engaging in the educational sessions. Three GPs did not attend the educational sessions: two of them prepared themselves by studying the training materials, whereas one indicated that the T2D-care in his practice was fully allocated to the PN and that he would only study the materials if needed.

*I prefer to choose whether I do a training or not based on my understanding of the theory. And if I get it, it is not needed. Foremost, I would simply like to send an email if I have a question. (GP5)*

All PNs followed at least one peer-to-peer session, and four out of seven PNs followed two sessions. Most of the HCPs were enthusiastic about the content of the training, which they found clear and helpful (mentioned both during the interviews and during the trainings). In general, HCPs mentioned that they did not consult the expert panel, because they found it unnecessary. As such, the expert panel was consulted twice. In each peer-to-peer session, HCPs brought forward challenging case studies they encountered during the implementation.

### Integration of the deprescribing programme in general practice

Most practices could not generate a list of eligible patients at the start of the study with the selection tool, even though most practices had experience with the use of similar tools, which delayed patient identification. In most practices, identification of eligible patients was eventually done together with the research assistant. Although the selection tool helped to identify eligible patients, the tool also retrieved many patients who were not eligible for deprescribing (Table 1). Two PNs mentioned that they did not prefer to use the selection tool, but instead would determine *ad hoc* if deprescribing was necessary. One PN indicated that she would prefer a digital warning in the individual patient file.

The help of the research assistant during the practice visit was highly appreciated by PNs:

*.… it was very clarifying when the research assistant came to the practice. Then you must do it [determine which patients are eligible for deprescribing], and you give yourself time to learn it. And that works. (PN6)*

One PN indicated that scheduling a practice visit with a research assistant created space in her busy agenda to focus on the deprescribing process. Another PN mentioned that although she understood the content of the training, she did not immediately know how to put that knowledge into practice. Many PNs also valued the research assistant's ability to perform administrative tasks.

Most HCPs found it challenging to assess the life expectancy and frailty of patients in order to determine HbA1c target values. In half the peer-to-peer sessions, this assessment was a point of discussion for PNs and GPs. Additionally, PNs noted in the interviews that patient involvement in the form of shared decision-making was challenging to integrate into day-to-day practice, as they were used to giving advice without elucidation of the patient perspective. One PN reported:

*It is the role we have as PN or GP to tell patients that it [the glucose values] is nice and low, we are going to reduce one of your pills. You do not need to explain it further. (PN6)*

Furthermore, three PNs and three GPs expressed their concerns about the size of the deprescribing steps, which were based on Dutch guidelines, and feared the need to restart medication. These concerns were justified by the data from the EMR, which showed that almost half (40%, Table 1) of the deprescribed patients (partially) restarted their glucose-lowering medication.

### The incorporation of study-related tasks within routine practice care

In general, PNs noted that the study-related tasks were too time-consuming to fit their primary care tasks. The indicated time needed to perform study tasks was variable and ranged from 4 h to two working days. The time needed to perform study-related tasks may also explain the low numbers of filled-out informed consent forms (*n* = 21) and filled-out questionnaires (first questionnaire *n* = 19 and second questionnaire *n* = 18, Table 1) retrieved from patients, and the low number of Mini-Cog^©^ examinations that were performed (first Mini-Cog^©^*n* = 22 and second Mini-Cog^©^*n* = 4, Table 1).

Most PNs indicated to have difficulties with asking for informed consent for study participation.

*… and then I have to approach somebody and ask for that informed consent. And, I did try that, for example on the phone, and then people want to know more about it and I do not know how to explain it. (PN6)*

Some PN’s indicated that it hindered the initiation of deprescribing of eligible patients. One GP was concerned that obtaining informed consent for study procedures could damage the patient-doctor relationship.

The Mini-Cog^©^ is a short examination to identify cognitive decline. PNs reported that they did not perform this measurement frequently since either they were unfamiliar with the Mini-Cog^©^ or they did not see the relevance of assessing cognitive decline in patients with hypoglycaemia. One PN noted that she associated hyperglycaemia, not hypoglycaemia, with dementia because it could indicate that the patient was forgetting to take medication. The HCPs who did perform the Mini-Cog^©^ examination reported that they liked doing the measurement because it only took a few minutes. They valued the insight into a patient's cognitive status.

Requesting a medication review by the pharmacist to improve the quality of the non-glucose-lowering medication was not part of the routine task of the PNs either. Most of the PNs indicated that they were never involved in medication reviews and were not experienced in delivering the necessary information to the pharmacy for the medication review.

#### Adjustments made for the randomized controlled trial

Results of the feasibility study were discussed with the research team and changes were made to the DPP (Table 3). In short, components of the DPP that improved the deprescribing process, such as educational sessions and practice visits, were made obligatory, whereas research aspects (e.g. obtaining informed consent) that interfered with the deprescribing process, such as asking for study participation, were allocated to the research assistant. Parts of the programme that did not align with routine care were adapted or made non-obligatory. Also, the PNs will be trained to address deprescribing during routine care without planning extra consultations.

**Table 3.** Adjustments made to the DPP for the OMED2 study.

Topic	DPP in feasibility study	DPP in RCT (OMED2 study)
DPP-content		
Educational sessions	Two obligatory educational sessions for PNs and GPs	Attending one educational session will be made obligatory for all HCPs, and a second educational session will be obligatory for PNs
Role of GP	Role of GP in deprescribing not discussed in educational sessions	Role of GP and the importance of the GP's expertise in deprescribing will be emphasized in the first educational session (GP important for assessing frailty, estimation of life expectancy, approving deprescribing)
Peer-to-peer sessions	At least one peer-to-peer session obligatory for PNs. GPs were invited to the peer-to-peer session but attendance was not obligatory	One peer-to-peer session will be obligatory for PNs, and PNs will be able to attend multiple peer-to-peer sessions
Patient consultation	PNs were asked to organize special consultations to address deprescribing	During the training, PNs will be advised to address the deprescribing during the routine consultations, which are organized every 3–6 months
Strategy to involve patient in decision-making	Strategy of patient involvement in the form of shared decision-making-based on literature [18]	Patient involvement will be in the form of HCPs recommending the patient to deprescribe with a clear explanation of why deprescribing is recommended with special attention to the patient's perspective
Deprescribing steps	Steps of deprescribing based on existing guideline [18, 19]	Education will be adapted to encourage deprescribing with smaller steps of dosage lowering
Patient selection tool	Patient selection tool used by PNs	For study purposes, the research assistants will generate a list of patients eligible for deprescribing with the selection tool during practice visits
Practice visits	Practice visits by research assistant when indicated as desired by HCPs	Two standard practice visits will be included in the DPP, with an optional third visit
Tasks to perform in daily general practice care		
Informed consent	Informed consent asked by PNs	Research assistants will send written informed consent via post to patients eligible for study participation
Medication review	For patients with many medications, HCPs were instructed to request a medication review	Medication review will no longer be obliged, but will be encouraged and explained during educational sessions and this information will be repeated during every peer-to-peer session
Mini-Cog^©^ examinations	Two Mini-Cog^©^ examinations performed in patients with deprescribing	Mini-Cog^©^ examinations will no longer be obliged, but will be encouraged and explained during educational sessions. In every peer-to-peer session, this information will be repeated

## Discussion

This study evaluated the feasibility of a glucose-lowering medication programme for overtreated elderly in general practice. Of the patients eligible for deprescribing, 86% were deprescribed, of whom 40% restarted their medication after deprescribing. DPP procedures and study tasks that were not part of regular care were perceived as time-constraining or inappropriate and hindered deprescribing. Practice visits by research assistants were perceived as helpful to facilitate implementation.

In this study, PNs were responsible for implementing the DPP, whereas most GPs indicated to be too busy to be involved. Previous studies in the Netherlands demonstrated that PNs can have a pivotal role in providing high-quality T2D-care [22, 23]. By design, however, these studies often excluded the more challenging T2D patients with comorbidities, which frequently accompany aging [24]. We observed that many GPs and PNs found it challenging to personalize HbA1c-reference values, which is instrumental in the decision to deprescribe. In addition, most HCPs felt that it was the role of the GP to decide on deprescribing. Therefore, it is important that the GP is also educated on deprescribing. For this reason, the DPP in the RCT will emphasize the role of the GP in deprescribing. It can be argued that, besides the GP, other HCPs, such as pharmacists, are important for deprescribing medication. Indeed, pharmacist-led interventions conducted in the Netherlands showed potential to increase deprescribing of medication in patients at risk of hypoglycaemia and/or low blood pressure, and this strategy was appreciated by pharmacists and patients [25–27]. Currently, a training programme for pharmacists is investigated to increase proactive and patient-centred deprescribing of medication [28].

The study also included clinical procedures and tasks for HCPs that may differ from current routine care, and these were found to be difficult to implement. Specifically, the communication strategy for patient involvement taught in the training (based on shared decision-making strategies used in pharmacy [18]) differed from how PNs communicated with patients. Shared decision-making implies that HCPs do not prefer a specific treatment when the options are closely balanced in terms of harms and benefits [29]. PNs indicated that they usually use more one-directional communication methods. In consultation with PNs and the study's patient council, we proposed an alternative strategy wherein the HCPs decide on the treatment and involve the patient in this decision.

Additionally, HCPs experienced difficulty in identifying patients eligible for deprescribing using the selection tool. A previous Dutch study on deprescribing cardiometabolic medication used a similar selection procedure to identify patients eligible for deprescribing. In this previous study, lists of eligible patients were created from data from the pharmacy, and the pharmacist decided, together with the GP and/or PN, which patients were eligible for deprescribing. Most pharmacists found this very useful to identify patients [25]. An alternative for identifying patients eligible for deprescribing may be using a reminder message in the EMR, which was perceived as useful by HCPs in a Canadian study on deprescribing proton pump inhibitors in general practice [30]. Nevertheless, we will allocate patient identification to the research assistant in the RCT, which has been proven useful in this feasibility study.

Finally, we found that many (40%) patients were restarted with their glucose-lowering medication after deprescribing. This indicates that the deprescribing steps we advised that were based on the textbook that was published by the main Dutch T2D training organization, were too big [i.e. decreasing 50% total daily insulin (TDI) or halving sulphonyl derivatives] [31]. Therefore, the DPP in the RCT recommends deprescribing with smaller steps (i.e. decreasing 25% TDI or halving sulphonyl urea derivatives).

### Limitations

A considerable advantage in the study was that we could evaluate and experiment with adjustments throughout the study. For instance, we found that administrative study tasks were frequently not performed by PNs and that allocating these tasks to the research assistant improved the number of patients who were deprescribed. Therefore, these tasks will be allocated to the research assistant in the RCT. A recent systematic review showed that this adaptation of an intervention to the target audience is an important attribute for scalability of the intervention [32].

A weakness of this study is its limited external validity due to the small number of practices participating and the national level of the study. Also, it can be argued that general practices that are willing to participate in a feasibility study are more motivated to improve diabetes care, which could be reflected in their perception of the use of the DPP.

## Conclusion

We studied the implementation of a DPP in general practice. Of the different strategies used, the education and peer-to-peer training sessions, together with the practice visits and the support of research assistants, were highly appreciated and seemed to stimulate implementation. Components of the programme that were not part of routine care, such as using the selection tool to identify patients and study tasks that HCPs needed to perform, were challenging to implement and hindered deprescribing.

## Supplementary Material

cmaf064_Supplementary_Data

## Data Availability

The data underlying this article will be shared on reasonable request to the corresponding author.
