# Supplementary material for: Feasibility of implementing an intervention in general practice for deprescribing of glucose-lowering medication in overtreated elderly
Source: Fam Pract. 2025 Aug 26;42(5):cmaf064. doi: 10.1093/fampra/cmaf064 (PMC12411905; doi:10.1093/fampra/cmaf064)
Supplement: cmaf064_Supplementary_Data [file cmaf064_supplementary_data.zip › FP_MS_Supplementary_material_July_2025.pdf]

SUPPLEMENTARY MATERIAL

Table S1 Topic list for semi-structured interviews with HCPs which illustrates the process of processing sensitizing concepts into questions. It was important that all topics were discussed during the interview. The phrasing and order of the questions could vary between interviews.

**Concept**	**Dimension**	**Topic**	**Questions**
**Capacity**	*Social roles*	Whether the HCP felt it was their role to perform the intervention	Q11 How do you see your role in performing this intervention?
		HCPs view on how patients perceive the role of the HCP in performing the intervention	Q12 How do you think patients perceived your role in this intervention?
		Expectations of HCPs towards the intervention	Q4 What were your expectations of this intervention?
	Social norms	Protocol for deprescribing diabetes medication	Q10 Did you follow the provided guideline exactly?
	Material resources	Explore if material resources were adequate to execute the intervention	Q20 What did you think about the supportive material?
		How much time was invested in implementing the intervention and what implications this would have on daily practice	Q26 How much extra time did you have to invest to perform this intervention?<br>Q26a To what extend can you maintain this in daily practice?
		If there was enough support to carry out the intervention	Q17 to what extend did you feel supported when performing this intervention?

			Q17a How do you see the support for this intervention in daily practice?
	Cognitive resources	Availability of sufficient knowledge of HCP	Q16 To what extend did you have sufficient background knowledge to perform the intervention?
		Quality of the offered educational sessions	Q18 What did you think of the educational sessions?
		Use of expert panel	Q19 Did you consult with the expert panel?<br>Q19a Why (not)?<br>Q19b How should the expert panel function in daily practice?
**Potential**	*Individual intentions*	Personal motivation to participate in this study	Q1 What was the reason to participate in this research?<br>Q2 How motivated were you to participate at the start of the intervention?<br>Q2a Did this change during the intervention?<br>Q3 How motivated are you to perform this intervention in the future?
	Shared commitment	Shared view of caregiver and patient on the intervention	Q13 What does the intervention mean to caregivers in your opinion?<br>Q14 What does the intervention mean to patients in your opinion?

		View of HCP on willingness of patient to follow the programme	Q15 To what extend did you feel patients were open to deprescribe their medication?
**Capability**	*Workability*	Encountered problems and solutions during the intervention	Q8 What problems did you encounter during this intervention?<br>Q8a What were the solutions for these problems?
		How the intervention was experienced by HCPs and patients	Q5 How did you experience participating in this intervention?<br>Q6 How do you think patients experienced this intervention?
		Specific moments when it was hard to follow the intervention	Q7 At what times was it difficult to follow the intervention?
	Integration	How the intervention worked out in day-to-day general practice	Q28 To what extend is it possible to carry out this intervention in daily practice?
		How the intervention worked out for patients	Q33 How did the intervention pan out for your patients?
**Contribution**	*Cognitive participation*	Opinion on collaboration between the different stakeholders (pharmacy, home care, nursing home doctors, specialists in the hospital, relatives)	Q21 How was the communication between you and the research group?<br>Q22 How was the communication between you and the patient?<br>Q23 How did the collaboration in the practice/ pharmacy go?

			Q25 What other stakeholders did you have to collaborate with to perform this research?<br>Q25a How did this collaboration pan out?
	Collective action	Distribution of tasks	Q24 How were the tasks divided in your practice?
		Extra work done to perform the intervention	Q27 Which actions did you have to take to put this intervention into practice?
		Trust in correctness of intervention	Q9 Did you doubt the correctness/completeness of the intervention?<br>Q9a If yes, what did you doubt?
	Reflexive monitoring	Reasons for recommending or discouraging colleagues to use the program	Q32 Why would you recommend or discourage the programme to your colleagues?
		Results of evaluation with colleagues about the intervention.	Q29 How did you evaluate the intervention in your practice?<br>Q29a What were the conclusions of the evaluation?
		Advantages for the practice yielded by the intervention	Q30 What did participation yield for your practice?
**Other**	*Future*	Future recommendations	Q31 How do you see implementing this intervention in a larger group in the future?

HCP: Healthcare provider
